# Effects of Internet and Smartphone Addictions on Depression and Anxiety Based on Propensity Score Matching Analysis

**DOI:** 10.3390/ijerph15050859

**Published:** 2018-04-25

**Authors:** Yeon-Jin Kim, Hye Min Jang, Youngjo Lee, Donghwan Lee, Dai-Jin Kim

**Affiliations:** 1 Department of Psychiatry, SMG-SNU Boramae Medical Center, Seoul 07061, Korea; loveaj1220@gmail.com; 2 Department of Statistics, Ewha Womans University, Seoul 03760, Korea; janghyemin23@naver.com; 3 Department of Statistics, Seoul National University, Seoul 08826, Korea; youngjo@snu.ac.kr; 4 Department of Psychiatry, Seoul St. Mary’s Hospital, The Catholic University of Korea College of Medicine, Seoul 06591, Korea

**Keywords:** anxiety, depression, Internet addiction, smartphone addiction, propensity score

## Abstract

The associations of Internet addiction (IA) and smartphone addiction (SA) with mental health problems have been widely studied. We investigated the effects of IA and SA on depression and anxiety while adjusting for sociodemographic variables. In this study, 4854 participants completed a cross-sectional web-based survey including socio-demographic items, the Korean Scale for Internet Addiction, the Smartphone Addiction Proneness Scale, and the subscales of the Symptom Checklist 90 Items-Revised. The participants were classified into IA, SA, and normal use (NU) groups. To reduce sampling bias, we applied the propensity score matching method based on genetics matching. The IA group showed an increased risk of depression (relative risk 1.207; *p* < 0.001) and anxiety (relative risk 1.264; *p* < 0.001) compared to NUs. The SA group also showed an increased risk of depression (relative risk 1.337; *p* < 0.001) and anxiety (relative risk 1.402; *p* < 0.001) compared to NCs. These findings show that both, IA and SA, exerted significant effects on depression and anxiety. Moreover, our findings showed that SA has a stronger relationship with depression and anxiety, stronger than IA, and emphasized the need for prevention and management policy of the excessive smartphone use.

## 1. Introduction

With the increasing use and convenience of the Internet and smartphones in daily life, the accumulated research has shown the negative effects of the excessive Internet and smartphone use in the realm of mental health [1].

The smartphone user rate in South Korean population is approximately 85%, the highest worldwide [2]. However, the excessive smartphone use is strongly associated with a number of mental health issues, including stress and an increased risk of abnormal anxiety [3,4]. Smartphone addiction (SA) has emerged as a new form of addiction along with Internet addictions (IA), and the clinical characteristic of the SA have received attention in recent years [5]. For example, there are some differences regarding the nature of the devices, such as the easy portability, real-time Internet access and direct communication features of smartphones [6]. Similarities and differences between IA and SA have been reported with respect to demographic variables and the motivational aspects of media use [1,6].

From the environmental aspect, a lack of alternative activities is associated with IA [7]. Additionally, being single has been reported to be strongly associated with both a social network and online gaming [8]. As to the educational level and monthly income dimensions, a recent study in people with SA found significant differences in the health dimension in favor of those who had a lower income and a lower degree of education [9]. Consistent with this finding, a systematic review reported significant correlation between academic performance and severity of IA [10]. With regard to age, a recent review found that problematic Internet use is most relevant to both adolescent and emerging adults (19 years and older) [10], while smartphone addiction is more prevalent in younger adolescents compared with emerging adults (19 years and older) [11]. A recent study showed that women tend to have a higher average of daily usage times and dependency scores for smartphones, compared to men [4]. Choi et al. (2015) reported that the male gender has a relevant risk factor for IA, and the female gender for SA [1]. Regarding the purpose of use, social networking showed to be more strongly related to a high smartphone dependence, compared to other mobile telephone-related functions [11]. In individuals with IA, Anderson et al. (2016) reported that male gender was significantly associated with online PC gaming [10]. 

With regard to psychological aspects, the positive associations of IA and SA with depression and anxiety have been widely reported [12,13]. Recent studies have suggested that addiction to the Internet and smartphones may arise by user’s individual cognitive-emotional and behavioral profile rather than the medium itself [14,15,16]. A recent study observed the role of empathy and life satisfaction in both IA and SA [17]. With regard to psychopathology, several studies reported a positive correlation between IA, depression, and anxiety [18,19,20], while a recent study reported a relationship between smartphone use and severity, depression, and anxiety [13]. Therefore, the interrelationship between IA, SA, and mental health problems needs to be precisely delineated. Moreover, given both the overlap and differences between IA and SA [16], then the question that arises is to what extent IA and SA are linked to the increased in the level of depression and anxiety after adjusting the confounding demographic and socioeconomic factors?

It remains unclear whether mental health problems are causes or consequences of excessive reliance on the Internet and smartphones. Cross-sectional studies have employed multiple regression analyses to investigate the relationships between mental health problems, IA, and SA in people [21]. However, in observational studies, which lack randomization, multiple regression analysis has limitations, such as the possibility of overestimation and a poor standard error when numerous covariates are present, in addition to the selection bias [22]. Thus, estimating the effects of addiction by simply examination of a particular outcome, such as depression and anxiety, would be biased by the imbalance of the demographic and socioeconomic factors associated with IA and SA. Moreover, no studies have yet investigated the differential effects according to the characteristics of Internet and smartphone users, including environmental contexts and users’ psychological profiles, of IA and SA on depression and anxiety. Propensity score matching (PSM) has become a popular approach to reduce the selection bias in observational studies [23,24]. In this paper, we applied PSM analysis to investigate the effects of IA and SA on depression and anxiety, in order to reduce the selection bias in our data. We chose sex, age, education, marital status, and income as confounding variable, considering the association of these sociodemographic variables with IA and SA in our study [9,25].

The primary aim of this study is to examine the interrelationships between IA, SA, and mood status, that is depression and anxiety, using propensity score matching analysis. Second, we seek to discover how the effects of depression and anxiety differ between IA and SA.

## 2. Materials and Methods

### 2.1. Study Participants

The data consisted of the online anonymous self-diagnosis survey responses of 5003 Korean adults (aged 19–49 years), conducted by the Catholic University of Korea, Seoul; and St. Mary’s Hospital in December 2014 [26]. The study was conducted in accordance with the Declaration of Helsinki. The institutional review boards of the Catholic University of Korea, Seoul; and St. Mary’s Hospital approved this study. All participants were informed about the study and provided written informed consent. The survey participants were recruited by a panel of a research company and self-report questionnaires were administered through Internet without any compensation. Only 149 respondents, who did not use smartphones, were excluded. Finally, we analyzed the data of 4854 participants. In the final sample, the ages were classified into three categories: Below 30 (33.19%), 30–39 (43.94%), and 40–49 (22.87%). There were 2573 males (53.01%) and 2281 females (46.99%). The additional demographic variables of participants considered were education, marital status, and income. 

### 2.2. Measures

#### 2.2.1. Measurement of Internet Addiction

The Korean Scale for Internet Addiction (K-scale) was developed in Korea to assess IA and has been validated in the Korean population with a high reliability of internal consistency [27]. The Cronbach’s alpha coefficient for the K-Scale was 0.91 [28]. It has seven subscales and 40 items, measuring daily life disturbance, disturbance of reality testing, automatic addictive thoughts, virtual interpersonal relationships, deviant behavior, withdrawal, and tolerance. This Likert type scale has been set from 1 (not at all) to 4 (always). According to the previous report using this scale, the participants were sorted into three groups: normal, potential risk, and high-risk [29]. The high-risk group was defined as having a standardized score of 70 or higher, in daily life disturbance, automatic addictive thoughts, tolerance factors, or at least 70 in total. The potential risk group was defined as a score of 62 or higher in daily life disturbance, automatic addictive thoughts, tolerance factors, or at least 63 in total. The normal use group contained those scores below these numbers. In this study, IA groups were made up of the potential risk and high-risk groups. 

#### 2.2.2. Measurement of Smartphone Addiction

The Smartphone Addiction Proneness Scale (K-SAS) has been validated and widely used to screen for SA [30]. It consists of 15 items rated in a four-point Likert type scale of distress from 1 (not at all) to 4 (always). The questions examined three factors: daily life disturbance, automatic addictive thoughts, and tolerance. The Cronbach’s alpha coefficient for the K-SAS was 0.880 [5].

Based on a previous report using this scale, we used the scores to classify the participants into three groups: Normal, potential risk, and high-risk [30]. The high-risk group was defined as having a score of 44 or more in total, or having a subscore of 15 or more in daily life disturbance along with subscores of 13 or more, in both automatic addictive thoughts and tolerance. The potential risk group was defined as having 41 or more in the total score, or 15 or more in the daily life disturbance factor. The normal use group contained those scores below these numbers [30]. In this study, the smartphone-addicted group were made up of high-risk and potential risk groups.

#### 2.2.3. Measurement of Mental Health Problems: Depression and Anxiety

The SCL-90-R is a multidimensional questionnaire developed to screen a range of psychological and psychopathological features of 9 subscales: Somatization, obsessive–compulsive, interpersonal sensitivity, depression, anxiety, hostility, phobic anxiety, paranoid ideation, and psychoticism [31]. The SCL-90 contains 90 items rated in a 5-point scale of distress from 0 (none) to 4 (extreme). The test–retest reliability of the SCL-90-R in the Korean language was 0.76 for depression and 0.77 for anxiety. The internal consistency was 0.89 for depression and 0.86 for anxiety [31]. Depression and anxiety have been reported to be the psychiatric symptoms most strongly associated with IA and SA [12,13]. The specific dimensions of interest to screen in this study included the SCL-90-R subscales for Depression and Anxiety.

### 2.3. Data Analysis

#### 2.3.1. Statistical Definition

Let $Z_{i}$ be a binary addiction indicator for the *i*th subject; that is, $Z_{i}=1$ if the *i*th subject is addicted (IA or SA), and $Z_{i}=0$ otherwise. The potential outcome of a mental problem (depression or anxiety) is defined as $Y_{i}\left(Z_{i}\right)$. Note that only one of the potential outcomes is observed at the same time for each subject, so direct computation of $Y_{i}\left(1\right)-Y_{i}\left(0\right)$ is impossible. Instead of the individual effect, the primary parameter of interest is the expected addiction effect on the addicted population

(1) $$\tau =E\left(Y_{i}\left(1\right)-Y_{i}\left(0\right)|Z_{i}=1\right)=E(Y_{i}\left(1\right)|Z_{i}=1)-E(Y_{i}\left(0\right)|Z_{i}=1)$$

However, the estimation of τ still has a problem because $E(Y_{i}\left(0\right)|Z_{i}=1)$ cannot be directly estimated. Of course, in randomized experiments, $E\left(Y_{i}\left(0\right)|Z_{i}=1\right)=E\left(Y_{i}\left(0\right)|Z_{i}=0\right)$ is satisfied, so τ can easily be estimated. However, in an observation study, the naïve estimation of τ can be biased because $E\left(Y_{i}\left(0\right)|Z_{i}=1\right)\neq E\left(Y_{i}\left(0\right)|Z_{i}=0\right)$. To adjust this selection bias, we assume that we can observe the covariates $X_{i}$ that are not affect by any addiction, and for a given covariates $X_{i},$ the potential outcomes $Y_{i}\left(1\right),\;Y_{i}\left(0\right)$ are conditionally independent of addiction indicator $Z_{i}.$ Furthermore, if potential outcomes are independent of the addiction conditional on covariates $X_{i}$, they are also independent of the addiction conditional in the propensity score $P\left(X_{i}\right)=\;P\left(Z_{i}=1|X_{i}\right)\;$[19]. The PSM estimator for τ becomes

(2) $$\tau^{PSM}=E_{P\left(X\right)|Z=1}\left[E\left(Y_{i}\left(1\right)|Z_{i}=1,\;P\left(X_{i}\right)\right)-E\left(Y_{i}\left(0\right)|Z_{i}=0,\;P\left(X_{i}\right)\right)\right]$$

#### 2.3.2. Estimating the Propensity Score

Propensity scores are calculated using logistic regression, a model used to predict the probability that an addiction occurs

(3) $$\log\frac{P\left(Z_{i}=1|X_{i}\right)}{1-P(Z_{i}=1|X_{i})}=\alpha +\;\beta^{T}X_{i}$$

In this paper, as the covariates for $X_{i}$, we consider five categorical covariates: sex (1 = male and 2 = female), age (1 = 20–29, 2 = 30–39, and 3 = 40–49), education (1 = middle school, 2 = high school, and 3 = university or above), marital status (1 = single, 2 = cohabitation, 3 = married, 4 = divorced, and 5 = bereaved), and income (1 = low, 2 = mid-low, 3 = middle, 4 = mid-high, and 5 = high). In Section 1, these covariates may influence simultaneously the outcomes (depression or anxiety) and addictions. Thus, for each subject, we estimated the propensity scores; that is, the conditional probability of being addicted given the observed covariates [32].

#### 2.3.3. Matching Methods Based on the Estimated Propensity Score

Once the propensity scores are estimated, matching can be used to estimate the treatment effect after adjusting to the differences between the two groups [33]. The goal of matching is to produce a matched sample that balances the distribution of a study’s patient and matched the covariates of the control groups observed. This adjusting method allows us to control the confounding variables. In this study, we adopted two widely used matching methods, the optimal and genetic matching [34].

#### 2.3.4. Estimation of the Relative Risks of Addiction on Mental Health Problems after Propensity Score Matching

After propensity score matching by using the observed covariates (age, gender, marriage, income, and education), we have a more balanced dataset. To model the mental health problem (depression or anxiety), we applied generalized linear models (GLMs) to the matched sample. Because the mental health scores are positive and biased, the gamma distribution with log link is fitted. Let $Y_{i}$ be an outcome of interest (an score of depression or anxiety) with mean $\mu_{i}$, we can use the Gamma GLM framework with covariates $X_{i}$:

$$\log\mu_{i}=\gamma^{T}X_{i}\;$$

Through modeling, we estimated $e^{\gamma }$ as the relative risks (as an expected mean difference between groups) of IA and SA for each covariate. 

## 3. Results

In addition to the 4854 participants, 126 (2.60%) were included in the IA group and 652 (13.43%) were included in the SA group. Table 1 shows the descriptive statistics of the depression and anxiety scores. The mean scores of depression and anxiety of IA and SA groups are larger than those of the normal use (NU) group. 

### 3.1. Matching Quality of the Propensity Score Matching Method

Although we condition only a few of the covariates in the questionnaires of this study, via the propensity score, we found that the matching procedure was sufficient to balance the distribution of each covariate, Table 2 and Table 3. We assessed the distances in the marginal distributions of $X_{i}$. For each covariate, we computed the bias; that is, the difference in sample averages of the addicted and normal samples. Before applying the propensity score matching, the biases were not ignored. However, after propensity score matching, the addiction and normal subsamples had a very similar marginal distribution for all covariates.

### 3.2. Effects of the Internet Addiction on Depression and Anxiety

The effects of IA on depression and anxiety obtained using propensity score matching are reported in Table 4. Through genetic matching, 3846 samples were selected. The IA was related to a greater risk of depression (relative risk 1.207, 95% confidence interval 1.128–1.292, and *p* < 0.001) and anxiety (relative risk 1.264, 95% confidence interval 1.173–1.362, and *p* < 0.001). All these relative risk ratios are significant because the confidence interval does not contain the 1. Through optimal matching, 252 samples were selected. The IA was related to a greater depression (relative risk 1.243, 95% confidence interval 1.145–1.348, and *p* < 0.001) and anxiety (relative risk 1.308, 95% confidence interval 1.192–1.435, and *p* < 0.001). Similar to the genetic matching, the relative risk ratios on both, depression and anxiety, are significantly larger than 1.

### 3.3. Effects of the Smartphone Addiction on Depression and Anxiety

The effects of SA on depression and anxiety using propensity score matching are reported in Table 4. Through genetic matching, 4516 samples were selected. The SA was related to a greater risk of depression (relative risk 1.337, 95% confidence interval 1.296–1.378, and *p* < 0.001) and anxiety (relative risk 1.402, 95% confidence interval 1.355–1.450, and *p* < 0.001). Through optimal matching, 1304 samples were selected. The SA was related to a greater risk of depression (relative risk 1.386, 95% confidence interval 1.334–1.440, and *p* < 0.001) and anxiety (relative risk 1.440, 95% confidence interval 1.380–1.503, and *p* < 0.001). All these relative risk ratios are significant.

### 3.4. Differences in Effects of the Internet and Smartphone Addiction on Depression and Anxiety

The relative risk ratios for depression and anxiety, from both genetic and optimal matching, were 10% higher for SA than for IA. This means that SA has a greater risk for depression and anxiety than IA. Those confidence intervals do not contain the 1, so we can say that SA is 34–44% more likely to cause a mental disorder.

## 4. Discussion

Our findings are that both IA and SA exert significant effects on depression and anxiety, even after controlling the confounders using propensity score matching. Epidemiological studies have estimated a higher prevalence of depression in IA [35,36]. A number of cross-sectional studies have reported that individuals with IA or SA showed higher levels of depression and anxiety than normal users [13,37]. In the present study, our results show the roles of IA and SA in developing depression and anxiety. There are some possible explanations for the current findings. First, addictive use of internet and smartphones can increase interpersonal problems, which is related to depression and anxiety, such as family conflicts, lack of off-line relationships, and a heightened need for approval in cyberspace. Second, withdrawal symptoms are proposed as psychopathological patterns in IA and SA, comparable to substance abuse disorders [5]. When they do not have access to a PC or smartphone, the individuals with IA or SA may become anxious, and then desire to use the Internet or a smartphone in to escape such negative feelings [38]. Another possible explanation is that unlike other addictive substances, such as alcohol and nicotine, internet and smartphones over-users may have little insight about their excessive use in daily life because of free and flexible access to the devices [3], making them experience their excessive use as an annoyance rather than as a sign of problematic behavior [39]. Another interesting finding was that SA exerted stronger effects on depression and anxiety than IA. This leads us to speculate that IA and SA have different influences on mental health problems. There could be several possible explanations for this finding. First, considering the media characteristics, it is easier for the excessive smartphone use develops through habit-forming nature of the device, because of its higher accessibility to the wireless network and 24 h of frequent notifications [39]. Second, with regard to environmental factors, this finding may reflect the current radical change of daily life average from PCs to smartphones. People may use the PC internet for complicated work and carry out the other daily tasks with smartphones, leading to a decrease in labor productivity and a higher level of stress [40]. Finally, individuals with SA may use smartphones to maintain relationships and a sense of connectedness with the online social network [41], leading to the fear of missing out and the fear of loss of connection, while triggering a higher smartphone use [42].

This study has several limitations to generalize findings to the entire population, such as the cross-sectional nature of the data limits and the interpretation of causal inference between the Internet and smartphone addiction, depression, and anxiety. Propensity matching also has limitations and requirements. The major limitation is that propensity scores can only control by observed confounders [43]. The possibility of unobserved confounders may remain, limiting the study finding for generalization. Furthermore, because of all observed confounders in this study were collected as categorical variables, there may be information loss when building PSM model. Therefore, our findings should be interpreted with caution. However, to get the robust results of matching, we considered two matching methods, genetic matching and optimal matching. Especially, genetic matching uses a genetic search algorithm, so its process can find a good matching solution with less loss of information [44]. Lastly, assessment of the depression and anxiety symptom was conducted by self-report psychological symptom measure using SCL-90-R. To evaluate mental health problems more accurately and consistently. A structured interview by clinician should be conducted in further studies.

## 5. Conclusions

In this study, we investigated how IA and SA influence mental health problems, depression and anxiety. To the best of our knowledge, this is the first study to estimate the association between IA, SA and psychopathology using propensity matching score method from cross-sectional data, and to investigate the differential effect in the psychopathology between IA and SA. In conclusion, our findings reveal that both IA and SA increase the risk of depression and anxiety. In addition, SA showed a stronger relationship with depression and anxiety compared to IA. 

An implication of these findings is that individuals with a problematic smartphone use should be closely monitored for mental health problems, highlighting the need to establish prevention and management policies aimed at the pre-clinical level of SA. Further prospective studies should investigate the causal directions of the relationships among IA, SA, and mental health problems and should identify the discriminative factors of IA and SA. 

## Figures and Tables

**Table 1:** Descriptive statistics of Depression and Anxiety scores.

Outcome	Statistics	Total (*n* = 4854)	Internet: NU (*n* = 4728)	IA (*n* = 126)	Smartphone: NU (*n* = 4202)	SA (*n* = 652)
**Depression**	Mean	26.69	26.52	33.01	25.49	34.42
	SD	10.3	10.23	10.7	9.55	11.48
	Min	13	13	13	13	13
	Max	65	65	62	65	65
	Skewness	0.74	0.76	0.29	0.74	0.34
**Anxiety**	Mean	18.47	18.33	23.75	17.51	24.67
	SD	7.79	7.7	9.21	7.04	9.37
	Min	10	10	10	10	10
	Max	50	50	50	50	50
	Skewness	1.02	1.03	0.56	1	0.47

Abbreviations: SD, standard deviation; NU, normal use; IA, Internet addiction; SA, smartphone addiction.

**Table 2:** Comparison of the mean percentage of baseline characteristics between IA and normal use groups, in the original sample and the propensity score matched sample, using the genetic and optimal matching.

	Before PSM			After PSM (Genetic)			After PSM (Optimal)		
	Normal (*n* = 4728)	IA (*n* = 126)	Bias	Normal (*n* = 3722)	IA (*n* = 124)	Bias	Normal (*n* = 126)	IA (*n* = 126)	Bias
Sex (male)	53.51	34.13	19.38	33.87	33.87	0	34.92	34.13	0.79
Sex (female)	46.49	65.87	−19.38	66.13	66.13	0	65.08	65.87	−0.79
Age (19–29)	33.1	36.51	−3.41	36.29	36.29	0	32.54	36.51	−3.97
Age (30–39)	43.99	42.06	1.93	42.74	42.74	0	44.44	42.06	2.38
Age (40–49)	22.91	21.43	1.48	20.97	20.97	0	23.02	21.43	1.59
Education (middle school)	0.59	0	0.59	0	0	0	0	0	0
Education (high school)	27.33	30.16	−2.83	30.65	30.65	0	37.3	30.16	7.14
Education (university or above)	72.08	69.84	2.24	69.35	69.35	0	62.7	69.84	−7.14
Marriage (single)	48.1	50	−1.9	50.81	50.81	0	43.65	50	−6.35
Marriage (cohabitation)	0.8	0.79	0.01	0	0	0	0.79	0.79	0
Marriage (married)	49.34	46.03	3.31	46.77	46.77	0	51.59	46.03	5.56
Marriage (divorced)	1.61	3.17	−1.56	2.42	2.42	0	3.97	3.17	0.8
Marriage (bereaved)	0.15	0	0.15	0	0	0	0	0	0
Income (low)	11.84	11.9	−0.06	12.1	12.1	0	14.29	11.9	2.39
Income (mid-low)	31.58	33.33	−1.75	33.06	33.06	0	34.13	33.33	0.8
Income (middle)	44.35	45.24	−0.89	45.97	45.97	0	42.06	45.24	−3.18
Income (mid-high)	10.89	7.14	3.75	6.45	6.45	0	7.14	7.14	0
Income (high)	1.33	2.38	−1.05	2.42	2.42	0	2.38	2.38	0

Abbreviations: PSM, propensity score matching; IA, internet addiction.

**Table 3:** Comparison of the mean percentage of baseline characteristics between SA and normal groups, in the original sample and the propensity score matched sample, using the genetic and optimal matching.

	Before PSM			After PSM (Genetic)			After PSM (Optimal)		
	Normal (*n* = 4202)	SA (*n* = 652)	Bias	Normal (*n* = 3873)	SA (*n* = 643)	Bias	Normal (*n* = 652)	SA (*n* = 652)	Bias
Sex (male)	55.45	37.27	18.18	36.86	36.86	0	36.5	37.27	−0.77
Sex (female)	44.55	62.73	−18.18	63.14	63.14	0	63.5	62.73	0.77
Age (19–29)	32.2	39.57	−7.37	39.5	39.5	0	39.26	39.57	−0.31
Age (30–39)	43.69	45.55	−1.86	45.72	45.72	0	45.86	45.55	0.31
Age (40–49)	24.11	14.88	9.23	14.77	14.77	0	14.88	14.88	0
Education (middle school)	0.55	0.77	−0.22	0.16	0.16	0	0.92	0.77	0.15
Education (high school)	27.44	27.15	0.29	27.22	27.22	0	28.68	27.15	1.53
Education (university or above)	72.01	72.09	−0.08	72.63	72.63	0	70.4	72.09	−1.69
Marriage (single)	47.52	52.15	−4.63	52.57	52.57	0	52.45	52.15	0.3
Marriage (cohabitation)	0.74	1.23	−0.49	0.93	0.93	0	0.61	1.23	−0.62
Marriage (married)	49.9	45.09	4.81	45.41	45.41	0	45.86	45.09	0.77
Marriage (divorced)	1.67	1.53	0.14	1.09	1.09	0	1.07	1.53	−0.46
Marriage (bereaved)	0.17	0	0.17	0	0	0	0	0	0
Income (low)	11.66	13.04	−1.38	12.75	12.75	0	13.5	13.04	0.46
Income (mid-low)	31.89	29.91	1.98	29.86	29.86	0	28.68	29.91	−1.23
Income (middle)	44.17	45.71	−1.54	46.35	46.35	0	46.01	45.71	0.3
Income (mid-high)	10.97	9.66	1.31	9.64	9.64	0	10.28	9.66	0.62
Income (high)	1.31	1.69	−0.38	1.4	1.4	0	1.53	1.69	−0.16

Abbreviations: PSM, propensity score matching; SA, smartphone addiction.

**Table 4:** Effects of the internet and smartphone addiction on depression and anxiety, based on propensity score matching.

		Internet Addiction			Smartphone Addiction		
Outcome	Type of PSM	*n*	RR	CI	*n*	RR	CI
Depression	Optimal	252	1.243	1.145–1.348	1304	1.386	1.334–1.440
	Genetic	3846	1.207	1.128–1.292	4516	1.337	1.296–1.378
Anxiety	Optimal	252	1.308	1.192–1.435	1304	1.44	1.380–1.503
	Genetic	3846	1.264	1.173–1.362	4516	1.402	1.355–1.450

Abbreviations: RR. relative risk; CI, confidence interval.
